# A multimodal deep learning model for predicting early neurological deterioration in patients with acute ischemic stroke

**DOI:** 10.3389/fneur.2026.1787921

**Published:** 2026-03-16

**Authors:** Chenglin Sun, Yaqiong Zhang, Peiyang Zhou, Zuneng Lu

**Affiliations:** 1 Department of Neurology, Renmin Hospital of Wuhan University, Wuhan, Hubei, China; 2 Xiangyang No.1 People’s Hospital, Hubei University of Medicine, Xiangyang, Hubei, China

**Keywords:** acute ischemic stroke, early neurological deterioration, multimodal machine learning, radiology reports, risk stratification

## Abstract

**Introduction:**

Timely identification of patients at high risk for early neurological deterioration (END) is critical for effective intervention after acute ischemic stroke; however, existing prediction models largely rely on structured clinical data and underutilize semantic information from imaging findings.

**Patients and methods:**

In this retrospective single-center study at Xiangyang No.1 People’s Hospital (January 2018–December 2023), 426 patients with acute ischemic stroke and imaging-confirmed middle cerebral artery occlusion who received non-endovascular treatment were included. Patients with other arterial occlusions, endovascular therapy, or incomplete data were excluded. END was defined as a ≥2-point increase in total National Institutes of Health Stroke Scale (NIHSS) score or a ≥1-point increase in the motor subscore within 7 days after admission. Structured clinical variables and radiology report text collected at admission were integrated into a multimodal deep learning model. Model performance was evaluated using AUC, accuracy, recall, precision, F1-score, calibration, and decision curve analysis, with interpretability assessed using SHAP and Integrated Gradients.

**Results:**

Among all patients, 38.0% exhibited END (30.3% early; 7.7% late). The multimodal Concat-Fusion model achieved AUCs of 0.877 (training) and 0.771 (test), surpassing single-modality models, with strong predictive capabilities for early (AUC = 0.842) and late END (AUC = 0.855). Subgroup analyses confirmed consistent performance across NIHSS scores, D-dimer levels, hypertension, and atrial fibrillation, with significantly higher AUC in diabetic patients (*p* = 0.002). SHAP analysis identified D-dimer, diastolic blood pressure, and heart rate as key contributors. Integrated Gradients revealed symptom descriptors including “unsteadiness,” “walking,” and “vision” as significant textual predictors. Risk stratification effectively distinguished high- and low-risk groups with significantly different cumulative END incidences within 48 h and 7 days (log-rank *p* < 0.0001). High-risk patients exhibited poorer NIHSS trajectories during 14-day follow-up.

**Conclusion:**

This multimodal prediction model may improve early identification of END and support individualized clinical management. Larger prospective studies are required to validate its clinical utility.

## 1 Introduction

Early neurological deterioration (END) denotes a quantifiable decline in neurological function during the acute phase of ischemic stroke, typically occurring within the initial days following symptom onset, and is significantly correlated with adverse clinical outcomes (1, 2). The predominant etiological factors contributing to END include symptomatic intracranial hemorrhage, malignant edema, seizures, and recurrent ischemic events (3–5). Nevertheless, approximately 50% of END cases lack a discernible cause, and the underlying mechanisms remain poorly understood (2, 3, 5, 6). Recent studies indicate that thrombus propagation (7), failure of collateral circulation (8), reperfusion injury (9, 10), and progressive cerebral edema may facilitate the development of END (11). Consequently, there currently exist no clinical guidelines for the prediction or management of END (12), complicating clinicians’ ability to identify high-risk patients in a timely manner for effective intervention during the critical period (e.g., 48 h to 7 days after hospital admission).

Despite growing interest in END prediction, existing models predominantly rely on structured clinical or laboratory variables in the electronic health record (EHR), such as demographic characteristics, vital signs, and laboratory biomarkers (13–15). While these variables provide valuable insights into a patient’s physiological condition, a considerable amount of unstructured clinical data, particularly radiology reports from CT or MRI examinations and descriptions of initial presenting symptoms, remains underutilized. Notably, radiology reports encompass rich semantic information regarding lesion location, morphology, vascular occlusion, and the status of surrounding tissues (16), which may be highly pertinent to END risk yet are not easily translatable into standard numerical variables. Previous studies have developed machine learning or natural language processing (NLP)-based models using radiology reports to identify stroke (17), predict stroke outcomes (16, 18), mortality (19), or stroke-related complications (20). However, these studies were not specifically designed for END prediction, and the integration of structured EHR data with radiology report text for END risk modeling has not been systematically explored.

Identifying patients with high END risk during the early phase may facilitate intensified monitoring, optimized hemodynamic management, individualized antithrombotic strategies, and timely preventive interventions, thereby potentially reducing neurological deterioration and improving short-term outcomes. Therefore, in this study, we developed a multimodal deep learning framework to predict the occurrence of END in patients with acute ischemic stroke. Specifically, our approach integrates structured clinical tabular data with unstructured radiology report text through NLP techniques, aiming to capture complementary information from both conventional clinical variables and deep semantic representations of imaging findings. We hypothesized that the multimodal model would outperform single-modality approaches and provide improved risk stratification for early clinical decision-making.

## 2 Methods

### 2.1 Study design and participants

This retrospective, single-center study was conducted at Xiangyang No.1 People’s Hospital between January 2018 and December 2023. Consecutive patients who met the following criteria were included: (1) diagnosed with acute ischemic stroke (within 48 h of onset) and confirmed to have middle cerebral artery occlusion as the culprit vessel based on neuroimaging; (2) received non-endovascular treatment after admission, including intravenous thrombolysis, antiplatelet therapy, and other standard medical management; and (3) had complete clinical records in diagnosis and follow-up data. Exclusion criteria included: (1) acute arterial occlusion not involving the middle cerebral artery; (2) receipt of endovascular treatment; (3) discontinuation of treatment after admission for any reason, resulting in inability to monitor neurological deterioration; (4) incomplete radiology reports or poor image quality (e.g., motion artifacts interfering with assessment); and (5) unavailable follow-up neurological assessments. Given the retrospective nature of this study, the requirement for informed consent was waived. The study protocol was approved by the Institutional Ethics Committee of Xiangyang No.1 People’s Hospital (Approval No. XYYYE20250017) and complied with the principles of the Declaration of Helsinki.

### 2.2 Data collection and definitions

A total of 426 patients were included in the final analysis. Baseline clinical characteristics, including demographic information, Glasgow Coma Scale (GCS) score, National Institutes of Health Stroke Scale (NIHSS) score, electrocardiogram (ECG) parameters, complete blood count, coagulation profile, initial biochemical tests, homocysteine (HCY), lipid profile, C-reactive protein (CRP), procalcitonin (PCT), interleukin-6 (IL-6), and cardiac biomarkers, were extracted from the EHR system. Text-based clinical information, including descriptions of initial presenting symptoms, ECG interpretations, echocardiography findings, major treatments, and documentation of the occlusion site, was also collected.

Follow-up neurological assessments were obtained from inpatient medical records and the follow-up system. NIHSS scores were recorded at 24 h, 48 h, 7 days, 15 days, 30 days, and 90 days after admission. These serial neurological and functional evaluations were used to determine early and late progression during follow-up. In addition, post-treatment laboratory tests were documented, including the first available results after treatment for complete blood count, coagulation profile, liver and renal function, lipid profile, cardiac enzymes, B-type natriuretic peptide (BNP), and CRP.

END was defined according to diagnostic criteria based on changes in the NIHSS score (21). Specifically, END was defined as a ≥ 2-point increase in the total NIHSS score or a ≥ 1-point increase in the motor subscore compared with the baseline NIHSS score assessed at admission. Neurological deterioration occurring within the first 48 h after symptom onset was classified as early END, whereas deterioration occurring between 48 h and 7 days was classified as late END.

### 2.3 Model construction and evaluation

Three predictive models were constructed to evaluate the contribution of different data modalities to the prediction of END, including (1) a clinical baseline model using structured tabular data; (2) a text baseline model using unstructured radiology report text; and (3) a multimodal fusion model integrating both data sources.

The clinical baseline model was developed using structured clinical variables extracted from the EHR. Prior to model construction, predictors with more than 40% missing values were excluded from the dataset. For lipid-related variables (e.g., total cholesterol and triglycerides) with missing pre-treatment values, the first available post-treatment measurements were used for deterministic imputation. For all remaining variables with missing values, multiple imputation was performed to reduce potential bias. Multiple machine learning algorithms, including random forest, LightGBM, XGBoost, and a fully connected neural network, were evaluated. Class imbalance in the training set was addressed using the Synthetic Minority Over-sampling Technique (SMOTE) (22). The model with the best performance on the validation set was selected as the final clinical baseline model. Meanwhile, the text baseline model was constructed using unstructured clinical text data. For text data, information from different textual sources was concatenated into a single input sequence. For missing text records, a fixed placeholder token (“Missing”) was inserted to ensure that all samples could be processed by the tokenizer and encoder. A pretrained medical-domain language model (MedBERT-KD Chinese) served as the backbone (23). Prior to model input, radiology reports were standardized to preserve clinically relevant semantic information while reducing noise. Text normalization included unifying full-width and half-width characters and removing extraneous whitespace, tab characters, line breaks, and non-informative symbols unrelated to clinical descriptions. The tokenizer associated with the pretrained MedBERT-KD Chinese model was used to process text input. Tokenization was performed following the WordPiece specification provided by the pretrained model. For Chinese text, tokenization was primarily performed at the character level, and special tokens (e.g., [CLS], [SEP], and [PAD]) were automatically handled using the default model configuration. Input sequences were truncated or padded to a fixed maximum length before being fed into the encoder. No stop-word removal was applied during model training, as transformer-based architectures rely on contextual attention mechanisms and are designed to process raw clinical text while preserving syntactic and semantic structure. During interpretability analysis, common function words and high-frequency non-specific clinical terms were filtered in the *post hoc* feature attribution stage to enhance readability and emphasize clinically meaningful textual predictors. The model parameters were fine-tuned on the END prediction task to derive contextual semantic representations.

**Figure 1:**
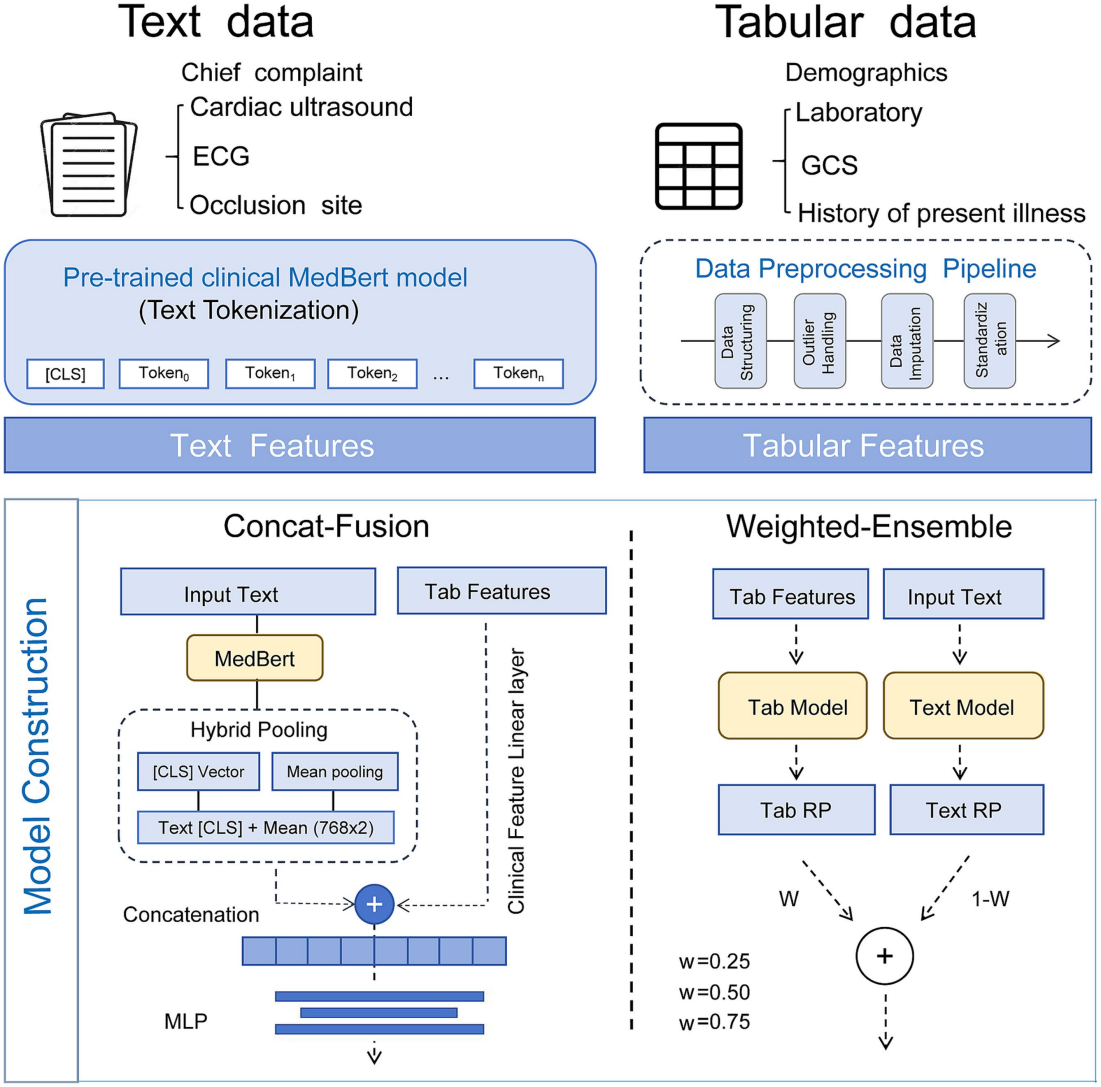
Model framework.

The multimodal fusion model was designed to combine complementary information from both structured and unstructured data (Figure 1). Two types of multimodal models were developed, including a concat-based fusion model (Fusion-Concat) and a weighted ensemble model (Weighted-Ensemble). In the Fusion-Concat model, clinical tabular features were first mapped through a fully connected transformation layer, while textual features were extracted using the MedBERT-KD Chinese encoder. To capture both global and fine-grained semantic information, a hybrid pooling strategy combining the [CLS] token embedding and the mean-pooled token representation was applied. The resulting two vectors were concatenated into a 1,536-dimensional text embedding (768 × 2), which was further concatenated with the mapped clinical features to form the multimodal representation. This fused vector was then passed through a multilayer perceptron for classification. In the Weighted-Ensemble model, predictions from the text-only and clinical-only baseline models were combined. After training both models independently, their output probabilities were linearly combined using the following equation:


$$P_{\text{fusion}}=w.P_{\text{text}}+(1-w)P_{\text{clinical}}$$

Three weighting coefficients (w = 0.25, 0.50, 0.75) were examined to evaluate the contribution of each modality.

Model training was implemented in PyTorch using the AdamW optimizer with a batch size of 8 for 15 epochs. To balance knowledge retention from the pretrained language model and adaptation to the downstream task, we adopted a differential learning rate strategy, setting a learning rate of 3 × 10^−5^ for the backbone text encoder and 6 × 10^−4^ for the classification head. A dropout rate of 0.3 and a weight decay of 1 × 10^−3^ were applied to reduce overfitting and improve generalization.

Model performance was assessed on an independent test set after splitting the dataset into training and test cohorts using a stratified 7:3 ratio to preserve the distribution of END and non-END cases. The primary evaluation metric was the area under the receiver operating characteristic curve (AUC), reported with 95% confidence intervals (CIs) to quantify discriminative ability. Multiple secondary metrics, including accuracy, recall (sensitivity), precision, and F1-score, were calculated to comprehensively evaluate classification performance. To assess agreement between predicted and observed risk, calibration curves were generated for each model, and the Brier score was computed as a quantitative measure of calibration quality. Clinical utility was further evaluated using decision curve analysis (DCA) across a range of clinically relevant threshold probabilities.

### 2.4 Interpretability analysis

Model interpretability was assessed separately for structured clinical features and unstructured text features. For clinical tabular variables, feature attribution was performed using SHapley Additive exPlanations (SHAP) to quantify the relative contribution of each variable to the model’s predictions. For text inputs, Integrated Gradients (IG) was applied at the embedding layer of the MedBERT-KD Chinese encoder to derive token-level attribution scores. Attribution values were then aggregated to the word level using Jieba segmentation, and global importance rankings were obtained by averaging attribution scores across all samples in the test set.

### 2.5 Statistical analysis

Categorical variables were summarized as counts and percentages and compared using the chi-square test or Fisher’s exact test, as appropriate. Continuous variables were expressed as mean ± standard deviation (SD) for normally distributed data or median (interquartile range, IQR) for non-normally distributed data, and compared using the independent-samples *t* test or the Wilcoxon rank-sum test, respectively. All statistical tests were two-sided. For subgroup analyses, model performance was evaluated across clinically relevant strata, including baseline NIHSS score (24), D-dimer level, and the presence of vascular risk factors such as hypertension, diabetes, or atrial fibrillation. An NIHSS score >4 was used to distinguish non-minor from minor stroke, consistent with commonly accepted clinical definitions (25). A D-dimer threshold of 2 mg/L was selected based on a previous report suggesting its association with increased risk of END (26). AUC and other performance metrics were calculated within each subgroup to assess model robustness and generalizability across heterogeneous patient populations. Risk stratification analysis was performed by applying the optimal cutoff determined from the Youden index (Youden index = sensitivity + specificity − 1) calculated from the receiver operating characteristic (ROC) curve in the training dataset (27). The threshold that maximized the Youden index was selected and subsequently applied to the independent test dataset. Patients with predicted probabilities greater than or equal to this threshold were classified as high-risk, whereas those below the threshold were classified as low-risk. The 95% CIs for all performance metrics were estimated using bootstrap resampling with 1,000 iterations. Kaplan–Meier survival curves were generated to compare the cumulative incidence of progressive stroke between groups within 3 and 7 days after onset. Differences between survival curves were assessed using the log-rank test. All statistical analyses were conducted using Python and R software packages. A *p*-value < 0.05 was considered statistically significant.

## 3 Results

### 3.1 Baseline characteristics

A total of 427 patients were screened for inclusion in the study, with one patient excluded due to incomplete follow-up, resulting in a final analysis cohort of 426 patients. The cohort exhibited a median age of 70.0 years, with 245 individuals (57.5%) identified as male. Of the total participants, 162 patients developed END. Patients in the END group had a significantly higher median NIHSS score on admission compared with the non-END group (12.0 [6.0–18.8] vs. 9.0 [4.0–15.0], *p* = 0.002). Additionally, initial measurements of systolic and diastolic blood pressure, respiratory rate, D-dimer, glucose levels, neutrophil count, and lactate dehydrogenase (LDH) levels were all significantly elevated in the END group (all *p* < 0.05) (Table 1; Supplementary Table 1).

**Table 1:** Baseline characteristics.

Characteristic	All patients (*n* = 426)	Non-END (*n* = 264)	END (*n* = 162)	*p* value
Age	70.0 [61.0, 79.0]	69.0 [59.0, 77.2]	70.0 [62.0, 80.0]	0.188
Sex (male, female)	245, 181	153, 111	92, 70	0.893
Initial systolic blood pressure	151.0 [137.0, 165.0]	150.0 [136.0, 160.0]	153.0 [138.2, 170.8]	0.018
Initial diastolic blood pressure	86.2 (14.9)	84.9 (13.5)	88.4 (16.7)	0.023
Respiratory rate (breaths/min)	20.0 [18.0, 20.0]	19.0 [17.0, 20.0]	20.0 [18.0, 21.0]	0.022
NIHSS score	10.0 [5.0, 17.0]	9.0 [4.0, 15.0]	12.0 [6.0, 18.8]	0.002
GCS total score	13.0 [10.0, 15.0]	13.0 [11.0, 15.0]	12.0 [9.0, 14.0]	0.01
Neutrophil count	5.9 [4.2, 7.7]	5.5 [4.1, 7.7]	6.2 [4.5, 7.9]	0.033
Neutrophil percentage	73.4 [65.1, 82.5]	71.6 [62.8, 81.6]	75.5 [67.5, 82.9]	0.013
Lymphocyte count	1.4 [1.0, 1.9]	1.4 [1.0, 2.0]	1.3 [1.0, 1.8]	0.285
Lymphocyte percentage	18.8 [11.4, 25.7]	19.6 [12.2, 27.0]	16.5 [10.8, 23.5]	0.017
D-Dimer	0.6 [0.3, 1.4]	0.5 [0.3, 1.0]	0.8 [0.4, 1.9]	<0.001
Glucose	7.0 [6.0, 8.8]	6.7 [5.8, 8.4]	7.2 [6.2, 9.5]	0.006
LDH	193.0 [167.0, 240.8]	185.5 [164.0, 234.0]	202.5 [170.3, 250.2]	0.017

END, early neurological deterioration; CT, computed tomography; NIHSS, National Institutes of Health Stroke Scale; GCS, Glasgow Coma Scale; LDH, lactate dehydrogenase. Data are presented as mean (SD) or median [Q1, Q3].

### 3.2 Performance of prediction models

**Figure 2:**
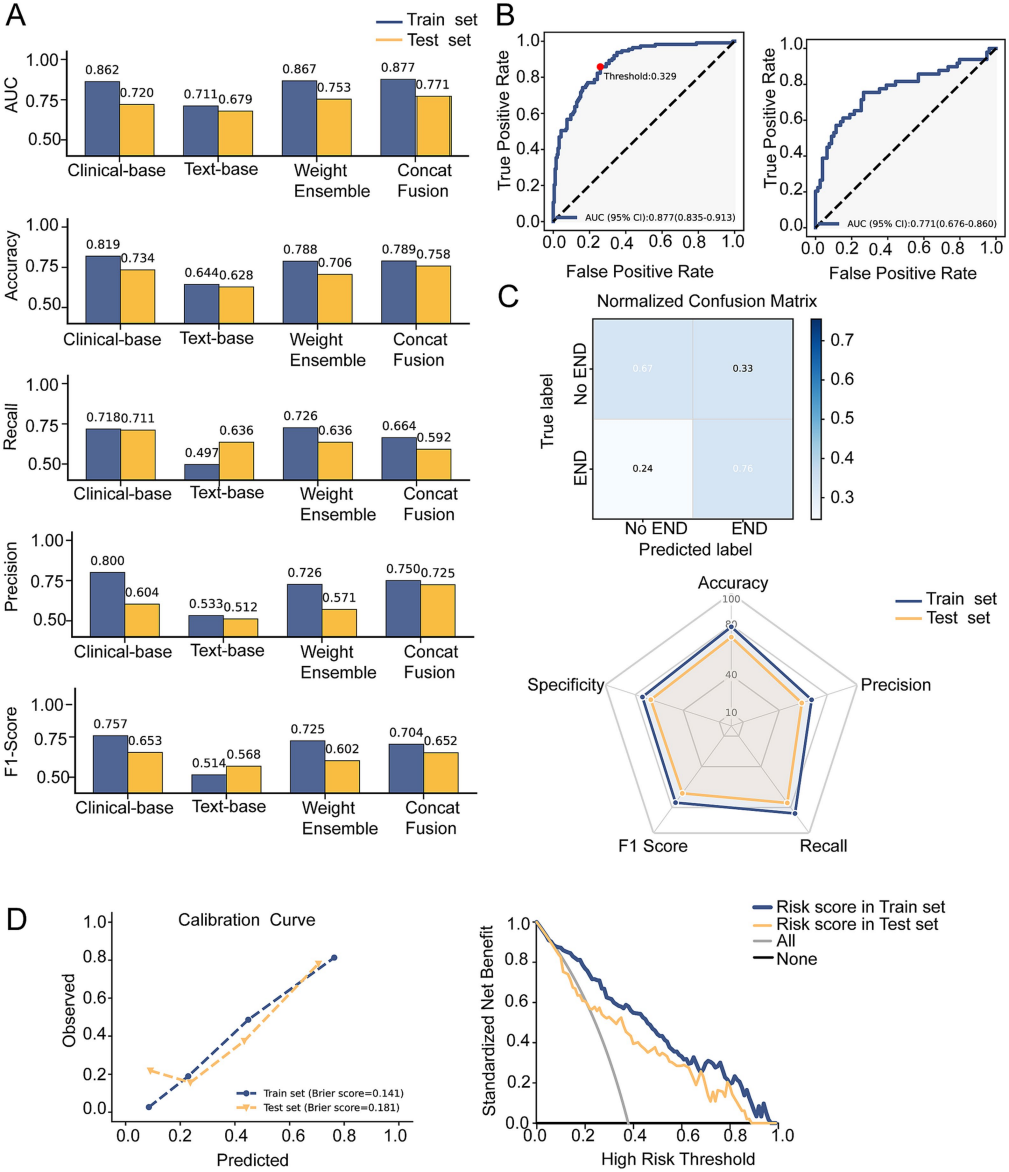
Performance of the models. **(A)** Area under the curve (AUC), accuracy, recall, precision, and F1-score of the four models on the training (blue) and test (orange) datasets. **(B)** Receiver operating characteristic (ROC) curves of the Concat-Fusion model on the training (left) and test (right) datasets. **(C)** Confusion matrix and performance of the Concat-Fusion model. **(D)** Calibration curve and decision curve of the Concat-Fusion model.

The clinical baseline model demonstrated superior discriminative ability compared with the text baseline model, achieving a higher AUC as well as better accuracy, recall, and F1-score in both the training and test datasets (Figure 2A; Tables 2, 3). The Concat-Fusion model achieved an AUC of 0.877 and 0.771 in the training and test datasets, respectively, outperforming the Weighted-Ensemble model and both single-modal baseline models (Figures 2A,B). The Weighted-Ensemble model also outperformed both single-modal models, with the optimal performance observed at a text-weight coefficient of w = 0.25. Using an optimal decision threshold of 0.329, the Concat-Fusion model achieved a true-positive rate of 0.76 and a true-negative rate of 0.67 in the test dataset (Figure 2C). In addition, the Concat-Fusion model demonstrated favorable calibration, with Brier scores of 0.141 and 0.181 in the training and test datasets, respectively (Figure 2D). The Concat-Fusion model was determined as the optimal model and was used in subsequent analyses.

**Table 2:** Performance of the models on the training dataset.

Model	AUC	Accuracy	Recall	Prec	F1
Clinical-base	0.862	0.819	0.718	0.800	0.757
Text-base	0.711	0.644	0.4966	0.533	0.514
Weighted-Ensemble	0.867	0.788	0.726	0.726	0.725
Concat-Fusion	0.877	0.789	0.664	0.750	0.704

AUC, area under the ROC curve; Accuracy, accuracy; Recall, sensitivity; Prec, precision; F1, F1-score.

**Table 3:** Performance of the models on the test dataset.

Model	AUC	Accuracy	Recall	Prec	F1
Clinical-base	0.720	0.734	0.711	0.604	0.653
Text-base	0.679	0.628	0.636	0.512	0.568
Weight Ensemble	0.753	0.706	0.636	0.571	0.602
Concat Fusion	0.771	0.758	0.592	0.725	0.652

AUC, area under the ROC curve; Accuracy, accuracy; Recall, sensitivity; Prec, precision; F1, F1-score.

### 3.3 Subgroup analysis

**Figure 3:**
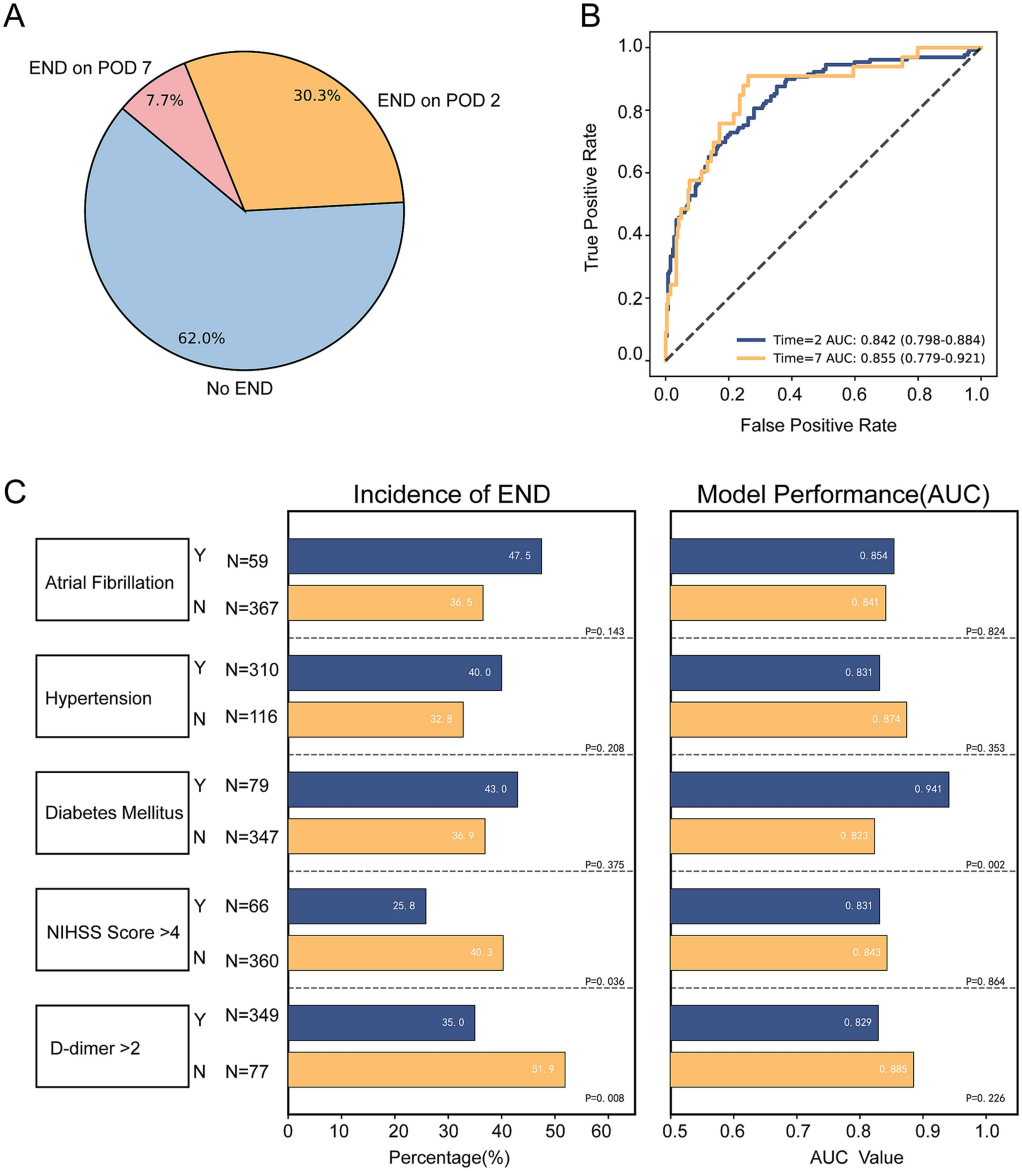
Subgroup analysis. **(A)** Proportion of patients with or without early neurological deterioration (END), including early and late END. **(B)** Receiver operating characteristic (ROC) curves of the Concat-Fusion model in patients with early or late END. **(C)** Performance of the Concat-Fusion model in different subgroups.

Among all included patients, 38.0% developed END, of whom 30.3% had early END and 7.7% had late END (Figure 3A). The Concat-Fusion model demonstrated strong discriminative ability for both early and late END, achieving AUCs of 0.842 (95% CI: 0.798–0.884) and 0.855 (95% CI: 0.779–0.921), respectively (Figure 3B). The model also maintained stable performance across multiple clinically relevant subgroups: comparable AUCs were observed among patients with NIHSS > 4 or D-dimer > 2 mg/L, with no statistically significant difference relative to their counterparts (*p* > 0.05) (Figure 3C). Similarly, model performance did not differ significantly between patients with or without atrial fibrillation or hypertension (*p* > 0.05). The model achieved a significantly higher AUC in patients with diabetes compared with those without (*p* = 0.002).

### 3.4 Interpretability analysis

**Figure 4:**
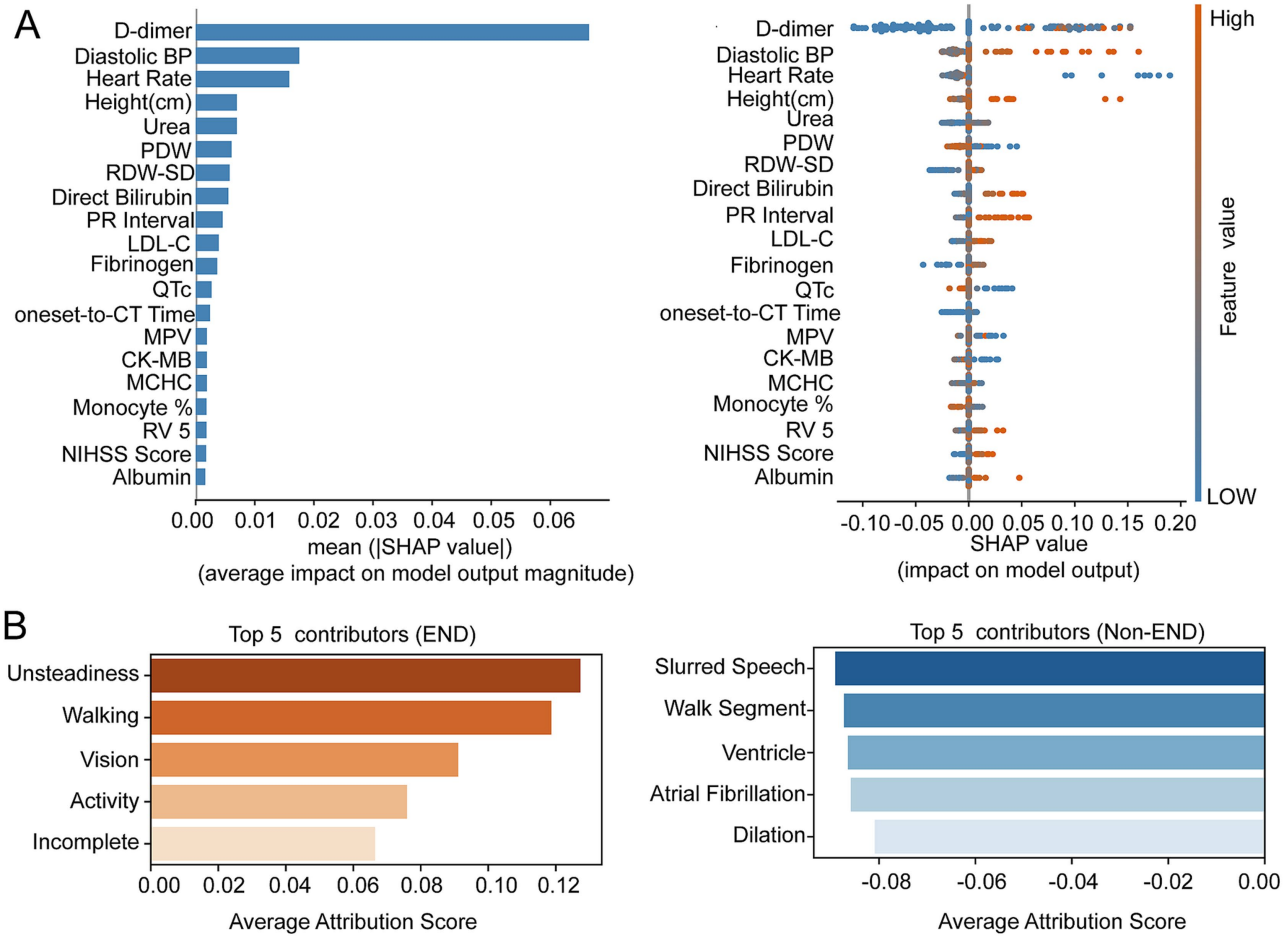
Interpretability analysis. **(A)** SHAP feature importance bar plot and beeswarm plot. **(B)** Top 5 contributing textual features identified by integrated gradients.

Feature attribution analyses were performed separately for structured tabular data and unstructured text data. SHAP analysis revealed that D-dimer level (mean SHAP score = 0.067), initial diastolic blood pressure (mean SHAP score = 0.018), and heart rate (mean SHAP score = 0.016) were the most influential predictors of END (Figure 4A). Furthermore, higher D-dimer levels exerted a prominent positive effect on the model output (Figure 4A). Abnormal variations in diastolic blood pressure and heart rate also contributed substantially to the model’s decision boundary. For the text features, words related to symptom instability, such as “unsteadiness” (mean IG score = 0.012), “walking” (mean IG score = 0.119), “vision” (mean IG score = 0.091), and “activity” (mean IG score = 0.076), were associated with higher predicted probabilities of END (Figure 4B). In contrast, terms such as “slurred speech” (mean IG score = −0.089), “wall segment” (mean IG score = −0.087), “ventricle” (mean IG score = −0.086), “atrial fibrillation” (mean IG score = −0.086), and “dilation” (mean IG score = −0.081), displayed negative attribution scores (Figure 4B).

### 3.5 Risk stratification and follow-up

**Figure 5:**
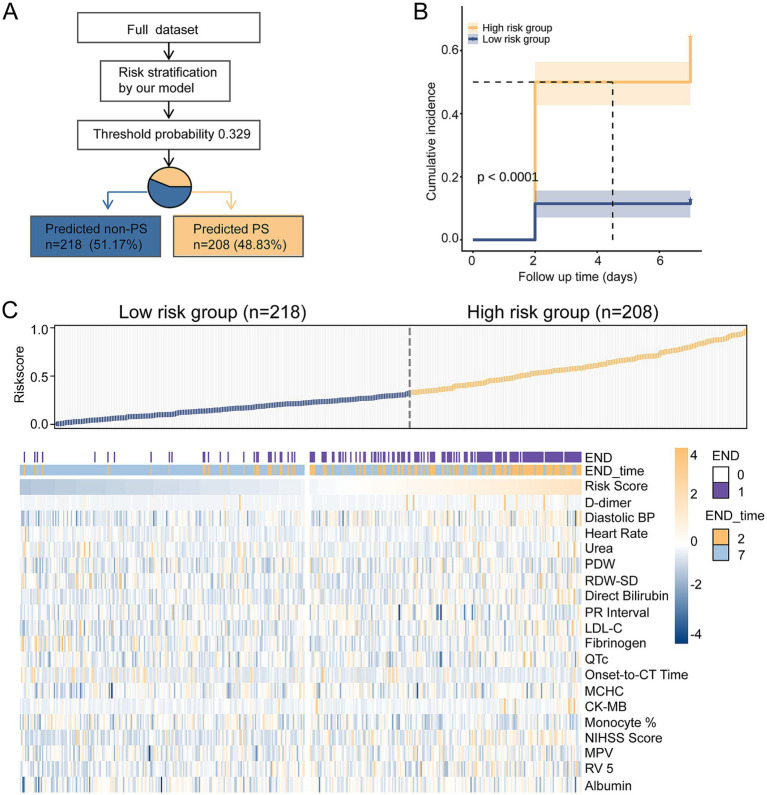
Risk stratification and distribution of characteristics. **(A)** Flowchart of the risk stratification process. **(B)** Kaplan–Meier curves showing the cumulative incidence of early neurological deterioration (END) in the high- and low-risk groups. **(C)** Heatmap of clinical characteristics of patients stratified by risk scores.

Based on the final multimodal model, the individualized risk score for END was generated for each patient. Using the optimal threshold of 0.329, the patients were stratified into two subgroups: a low-risk group (predicted non-END, *n* = 218, 51.17%) and a high-risk group (predicted END, *n* = 208, 48.83%) (Figure 5A). In the low-risk group, 28 patients (12.84%) developed END, whereas 190 patients (87.16%) did not. In contrast, in the high-risk group, 134 patients (64.42%) developed END and 74 patients (35.58%) did not. Univariate Cox proportional hazards analysis demonstrated that, compared with the low-risk group, patients in the high-risk group had a significantly increased risk of progressive stroke (hazard ratio [HR] = 7.20, 95% CI: 4.78–10.83, *p* < 0.0001). When analyzed as a continuous variable, each 5% increase in the predicted risk score was associated with a 21% increase in the hazard of progressive stroke (HR = 1.21, 95% CI: 1.17–1.24, *p* < 0.0001) (Supplementary Figure 1). Patients in the high-risk group exhibited a significantly higher cumulative incidence of END within both 48 h and 7 days, whereas the low-risk group maintained a consistently low event rate over the same intervals (log-rank *p* < 0.0001) (Figure 5B). The feature heatmap further revealed that END cases were strongly concentrated within the high-risk region, whereas non-END were predominantly located in the low-risk region (Figure 5C). Furthermore, patients with early END were most concentrated in the highest-risk region. Higher D-dimer and initial diastolic blood pressure also exhibited clustering in the high-risk region.

**Figure 6:**
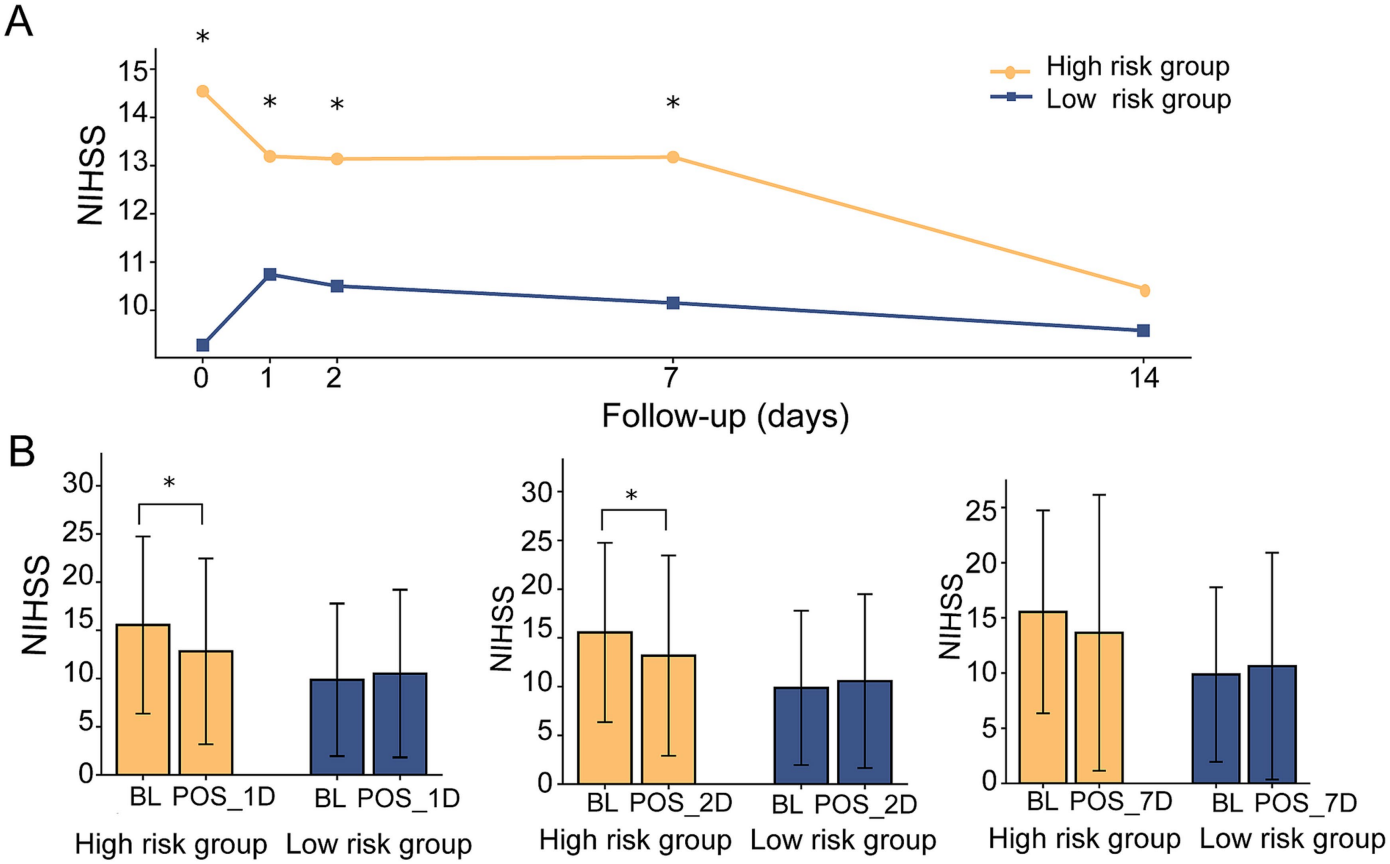
Follow-up NIHSS scores. **(A)** Trajectories of NIHSS scores in the high- and low-risk groups during follow-up. **(B)** Comparison of NIHSS scores at baseline (BL) and at 1, 2, and 7 days after treatment.

During the 14-day follow-up, both groups demonstrated gradual improvement after treatment and showed declining NIHSS scores. However, the high-risk group consistently exhibited higher NIHSS scores on day 1 (*p* = 0.012), day 2 (*p* = 0.036), and day 7 (*p* = 0.071, borderline significance) compared with the low-risk group (Figure 6A). Compared with the baseline, the high-risk group had significantly lower NIHSS scores at day 1 and day 2 after treatment (Figure 6B). In contrast, no significant differences were observed between baseline and post-treatment NIHSS scores in the low-risk group.

## 4 Discussion

In this study, we developed a multimodal deep learning model that integrates clinical variables with radiology report text to predict END in patients with acute ischemic stroke. Our model demonstrated better discriminative performance compared with single-modal approaches and remained robust across multiple clinical subgroups. Furthermore, the predicted risk score enabled effective stratification of patients into high- and low-risk groups, which showed significantly different short-term END incidence and follow-up neurological outcomes. These findings suggest that incorporating semantic information from radiology reports could enhance END prediction and may support earlier monitoring and individualized clinical management.

Previous research on predicting END in acute ischemic stroke mainly utilized readily available structured clinical or laboratory predictors, such as baseline NIHSS score, inflammatory or coagulation biomarkers, vascular risk factors, or simple demographic and comorbidity data (28–30). These models, often developed using logistic regression or nomogram approaches, have reported AUCs ranging from approximately 0.72 to 0.85 (28, 30). In our study, the clinical data-based model demonstrated comparable performance, achieving AUCs of 0.862 and 0.720 in the training and test sets, respectively. Conversely, imaging-based prediction models using MRI, CT, or transcranial Doppler ultrasound have primarily focused on predicting overall stroke outcomes (31–33). Some multimodal radiomics models integrating imaging and clinical features have reported AUCs approaching 0.90 (31, 33); however, these studies were primarily designed to predict functional outcomes rather than END occurrence. In contrast, our study utilized text derived from radiology reports rather than raw imaging data, and the text-only model achieved AUCs of 0.711 and 0.679 in the training and test datasets, respectively. This relatively lower performance may reflect the fact that radiology reports represent a summarized interpretation of imaging findings and may not fully capture the detailed spatial and quantitative information available in raw imaging data. Several recent studies have also applied NLP models to process radiology reports to detect complications of ischemic stroke or predict functional outcomes (18, 20, 34). However, most of these studies have failed to integrate clinical data and imaging or textual data into a unified predictive framework and were not designed to predict END specifically. Our study expands this paradigm by combining structured EHR data with semantic information extracted from radiology report text using a multimodal deep-learning framework, resulting in substantially improved predictive performance and risk stratification, with maximum AUCs reaching 0.877 and 0.771 in the training and test sets, respectively. These findings suggest that integrating structured clinical data with textual information may provide additional value over single-modality approaches for END prediction. Further direct comparison with existing END prediction models would help more precisely define the relative performance of our approach and will be explored in future work.

Textual information extracted from radiology reports offers several advantages for predictive models in acute stroke. Unlike raw imaging data, radiology reports represent a distilled and clinically meaningful interpretation that has already undergone expert synthesis, allowing the model to leverage high-level semantic cues, such as lesion description, vascular status, and early pathological changes, that may not be easily captured from pixel-level features alone. In addition, processing text requires substantially less computational overhead than analyzing 3-dimensional imaging data, making NLP-based models easier to train, deploy, and integrate into routine clinical workflows. However, reliance on textual reports also introduces important limitations. Model performance is inherently dependent on the quality and completeness of radiology documentation; inconsistent reporting styles or suboptimal interpretations may introduce noise that cannot be corrected by the model. Furthermore, despite using a medical-domain pretrained language model in this study, current Chinese medical NLP tools remain limited by imperfect tokenization and domain-specific vocabulary handling, which may restrict the model’s ability to fully capture important clinical semantics. For example, the key words identified in our study included terms such as “wall segment,” “ventricle,” “atrial fibrillation,” and “dilation,” which are clinically relevant but not directly specific to END, reflecting the limitations of current medical language models in capturing task-specific nuances. Continued improvement in standardized reporting practices and medical language models will be essential to further enhance the reliability of text-based prediction systems.

Our multimodal model offers a practical tool to flag high-risk individuals during the vulnerable first 48 h to 7 days, enabling prompt intensified monitoring, haemodynamic stabilization, or tailored antithrombotic/anticoagulant strategies, which may reduce the risk or severity of END (1). From a clinical workflow perspective, the proposed model could be implemented as an automated decision-support tool embedded within the hospital EHR system. Upon admission, structured clinical variables and radiology report text are routinely generated, allowing real-time risk estimation without additional data collection burden. Patients identified as high-risk could be considered for intensified neurological monitoring during the first 48 h, closer hemodynamic surveillance, early repeat imaging, or tailored antithrombotic management. Furthermore, early risk stratification may assist clinicians in resource allocation, such as prioritizing high-risk patients for the stroke unit or intensive care observation. Nevertheless, it should be noted that although we incorporated interpretability analyses to enhance model transparency, prospective validation and real-world performance evaluation are necessary to further build clinical trust. Moreover, implementation of AI-based decision-support systems requires careful consideration of data privacy, algorithmic fairness, accountability, and integration within existing EHR systems before routine clinical deployment. From a fundamental research perspective, by revealing which clinical and semantic features (e.g., coagulation markers, blood pressure instability, lesion descriptors) most strongly drive risk, the model may help uncover underlying mechanisms of END and shed light on its unknown etiology, thereby proposing new preventive targets.

This study has several limitations. Firstly, the generalizability of our model may be influenced by patient demographics, healthcare system characteristics, and radiology reporting practices. This was a retrospective study conducted in a single tertiary center with a relatively homogeneous patient population and standardized reporting formats. Hence, variations in stroke subtype distribution, comorbidity profiles, potential confounding factors not included in our dataset, and treatment protocols in other institutions or countries may affect model performance. Exclusion of patients with non-middle cerebral artery occlusions or incomplete clinical and radiological data may have introduced selection bias and limited the representativeness of the study population, potentially affecting the external applicability of the findings. Furthermore, variability in the quality and consistency of radiology reports may introduce additional uncertainty. In addition, the NLP component was developed using a Chinese medical language model, and its applicability to radiology reports written in other languages or formats may require adaptation or retraining. Therefore, external multicenter validation across diverse healthcare settings is essential before broader clinical implementation. Secondly, although we used a medical-domain pretrained language model, current Chinese NLP tools still have limitations in tokenization and domain-specific semantic representation, which may restrict the model’s ability to fully capture imaging descriptions. Finally, we did not incorporate raw imaging data, such as CT or MRI, which may contain additional information complementary to textual and clinical features. Future studies integrating multimodal imaging, prospective validation, and real-world deployment are warranted.

## 5 Conclusion

We developed and validated a multimodal deep learning model integrating structured clinical variables and radiology report text to predict END in patients with acute ischemic stroke. The multimodal approach demonstrated improved discriminative performance compared with single-modality models and enabled effective risk stratification. By facilitating early identification of high-risk patients, the model may support intensified monitoring and individualized management during the critical early phase after stroke. However, given the retrospective single-center design, external multicenter validation and prospective impact assessment are required. Future studies should focus on independent benchmarking, real-world implementation, and evaluation of clinical decision-making outcomes.

## Data Availability

The original contributions presented in the study are included in the article/Supplementary material, further inquiries can be directed to the corresponding authors.

## Supplementary material

SUPPLEMENTARY FIGURE 1 Univariate Cox regression analysis.
