# Supplementary figures and images for: A multimodal deep learning model for predicting early neurological deterioration in patients with acute ischemic stroke
Source: Front Neurol. 2026 Mar 16;17:1787921. doi: 10.3389/fneur.2026.1787921 (PMC13033536; doi:10.3389/fneur.2026.1787921)

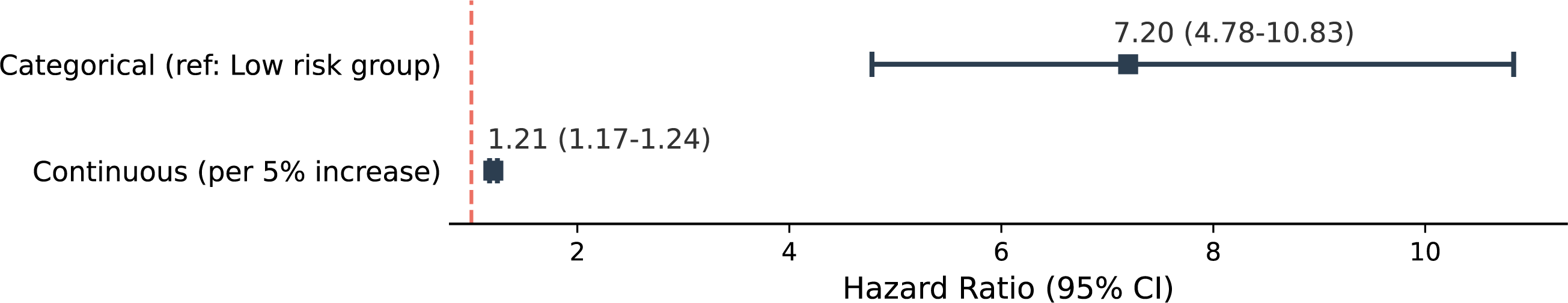

Supplement: SUPPLEMENTARY FIGURE 1 — Univariate Cox regression analysis. [file Image_1.tif]
