# Supplementary material for: A multimodal deep learning model for predicting early neurological deterioration in patients with acute ischemic stroke
Source: Front Neurol. 2026 Mar 16;17:1787921. doi: 10.3389/fneur.2026.1787921 (PMC13033536; doi:10.3389/fneur.2026.1787921)
Supplement: Supplementary file 2 [file Table_1.docx]

Supplementary Material

# Supplementary Table S1.

Additional baseline characteristics

|  |  | All patients  (n=426) | Non-END  (n=264) | END  (n=162) | P value |
| --- | --- | --- | --- | --- | --- |
| Height (cm) |  | 165.0 [158.2,170.0] | 165.0 [160.0,170.0] | 165.0 [158.0,170.0] | 0.818 |
| Weight (kg) |  | 63.0 [57.2,72.0] | 65.0 [60.0,72.0] | 60.0 [55.0,70.0] | 0.124 |
| Heart rate (bpm) |  | 78.5 [68.2,95.8] | 77.0 [68.0,94.0] | 81.0 [69.2,97.0] | 0.316 |
| Onset-to-CT time |  | 5.0 [2.0,12.0] | 4.8 [2.0,12.0] | 5.0 [2.5,12.0] | 0.63 |
| Hypertension | Y | 310 (72.8) | 186 (70.5) | 124 (76.5) | 0.208 |
|  | N | 116 (27.2) | 78 (29.5) | 38 (23.5) |  |
| Diabetes | Y | 79 (18.5) | 45 (17.0) | 34 (21.0) | 0.375 |
|  | N | 347 (81.5) | 219 (83.0) | 128 (79.0) |  |
| Coronary artery disease | Y | 93 (21.8) | 56 (21.2) | 37 (22.8) | 0.784 |
|  | N | 333 (78.2) | 208 (78.8) | 125 (77.2) |  |
| Atrial fibrillation | Y | 59 (13.8) | 31 (11.7) | 28 (17.3) | 0.143 |
|  | N | 367 (86.2) | 233 (88.3) | 134 (82.7) |  |
| Smoking | Y | 131 (30.8) | 83 (31.4) | 48 (29.6) | 0.776 |
|  | N | 295 (69.2) | 181 (68.6) | 114 (70.4) |  |
| Drinking | Y | 149 (35.0) | 92 (34.8) | 57 (35.2) | 1 |
|  | N | 277 (65.0) | 172 (65.2) | 105 (64.8) |  |
| Heart rate |  | 78.0 [70.0,88.0] | 78.0 [70.0,87.0] | 78.5 [70.0,90.0] | 0.23 |
| P-R interval (ms) |  | 159.5 [148.0,174.0] | 159.0 [148.0,171.2] | 162.0 [148.0,175.8] | 0.434 |
| QRS duration (ms) |  | 92.0 [87.0,99.0] | 92.0 [87.0,100.0] | 92.0 [86.0,98.8] | 0.552 |
| QT interval (ms) |  | 384.0 [354.0,414.8] | 386.0 [357.0,414.2] | 381.0 [351.0,414.0] | 0.253 |
| Corrected QT interval |  | 441.0 [422.0,463.0] | 441.0 [422.0,461.0] | 442.0 [423.5,465.0] | 0.591 |
| Electrical axis |  | 31.0 [3.0,58.0] | 29.0 [3.0,58.0] | 32.5 [0.5,57.0] | 0.843 |
| SV1 |  | 0.7 [0.4,1.1] | 0.7 [0.4,1.1] | 0.7 [0.4,1.1] | 0.964 |
| RV5 |  | 1.5 [1.1,2.0] | 1.5 [1.1,1.9] | 1.7 [1.1,2.2] | 0.057 |
| RV5+SV1 |  | 2.3 [1.7,3.0] | 2.3 [1.6,2.9] | 2.4 [1.8,3.1] | 0.125 |
| WBC |  | 8.0 [6.4,9.9] | 8.0 [6.3,9.8] | 8.1 [7.0,10.0] | 0.236 |
| RBC |  | 4.4 [4.0,4.8] | 4.4 [4.0,4.8] | 4.4 [4.0,4.8] | 0.673 |
| Hb |  | 135.5 [123.0,150.0] | 136.0 [123.0,149.2] | 135.0 [123.0,150.0] | 0.836 |
| HCT |  | 0.4 [0.4,0.4] | 0.4 [0.4,0.4] | 0.4 [0.4,0.4] | 0.999 |
| MCV |  | 91.8 [88.8,95.2] | 92.0 [88.8,95.6] | 91.2 [88.6,94.3] | 0.125 |
| MCH |  | 31.0 [29.7,32.2] | 31.2 [29.8,32.1] | 30.9 [29.4,32.2] | 0.168 |
| MCHC |  | 336.0 [329.0,343.0] | 336.0 [330.0,343.0] | 337.0 [327.0,344.0] | 0.849 |
| RDW-CV |  | 13.1 [12.6,13.6] | 13.0 [12.5,13.6] | 13.2 [12.7,13.7] | 0.039 |
| RDW-SD |  | 43.8 [41.5,46.3] | 43.8 [41.1,46.1] | 43.7 [41.8,46.4] | 0.553 |
| PLT |  | 208.5 [167.0,243.0] | 209.0 [164.0,243.0] | 207.0 [172.2,245.5] | 0.725 |
| PCT |  | 0.2 [0.2,0.3] | 0.2 [0.2,0.3] | 0.2 [0.2,0.3] | 0.319 |
| MPV |  | 10.7 [9.9,11.7] | 10.7 [9.9,11.5] | 10.8 [10.0,11.8] | 0.459 |
| PDW |  | 13.7 [11.7,16.2] | 13.7 [11.7,16.2] | 13.6 [11.6,16.3] | 0.698 |
| P-LCR |  | 30.8 [25.4,38.3] | 30.8 [25.0,37.6] | 31.1 [25.4,38.4] | 0.546 |
| Monocyte count |  | 0.5 [0.4,0.6] | 0.5 [0.4,0.6] | 0.5 [0.4,0.6] | 0.781 |
| Monocyte percentage |  | 6.3 (2.2) | 6.4 (2.3) | 6.1 (2.0) | 0.178 |
| Eosinophil count |  | 0.1 [0.0,0.1] | 0.1 [0.0,0.1] | 0.1 [0.0,0.1] | 0.138 |
| Eosinophil percentage |  | 0.8 [0.2,1.5] | 0.8 [0.2,1.5] | 0.6 [0.1,1.5] | 0.137 |
| Basophil count |  | 0.0 [0.0,0.0] | 0.0 [0.0,0.0] | 0.0 [0.0,0.0] | 0.944 |
| Basophil percentage |  | 0.3 [0.2,0.5] | 0.3 [0.2,0.5] | 0.3 [0.2,0.5] | 0.623 |
| PT |  | 10.9 [10.3,11.7] | 10.9 [10.3,11.6] | 11.0 [10.3,11.7] | 0.222 |
| PTR |  | 104.1 (22.8) | 105.0 (21.5) | 102.7 (24.8) | 0.336 |
| INR |  | 0.9 [0.9,1.0] | 0.9 [0.9,1.0] | 0.9 [0.9,1.0] | 0.397 |
| APTT |  | 25.1 [22.3,27.7] | 25.1 [22.1,27.8] | 25.1 [22.4,27.6] | 0.85 |
| TT |  | 18.3 [17.1,19.4] | 18.3 [17.2,19.4] | 18.3 [17.1,19.4] | 0.954 |
| Fbg |  | 2.8 [2.3,3.4] | 2.7 [2.3,3.4] | 2.8 [2.4,3.4] | 0.359 |
| ALT |  | 16.9 [11.9,23.3] | 17.2 [11.9,23.7] | 16.2 [11.9,21.2] | 0.257 |
| AST |  | 29.1 [23.4,37.5] | 28.6 [23.6,36.2] | 30.2 [23.3,38.4] | 0.588 |
| GGT |  | 22.8 [15.9,34.1] | 21.7 [15.8,33.6] | 24.8 [16.3,36.0] | 0.393 |
| ALP |  | 77.1 [64.0,93.7] | 74.3 [63.3,92.8] | 80.0 [65.4,95.1] | 0.142 |
| Total bilirubin |  | 15.2 [11.8,20.4] | 14.9 [11.7,19.4] | 16.2 [12.0,21.3] | 0.102 |
| Direct bilirubin |  | 4.6 [3.6,6.2] | 4.5 [3.5,6.1] | 4.8 [3.7,6.6] | 0.179 |
| Indirect bilirubin |  | 10.8 [7.8,14.4] | 10.6 [7.4,14.0] | 11.5 [8.1,15.0] | 0.146 |
| Total protein |  | 68.2 [64.8,72.3] | 67.9 [64.1,72.4] | 68.9 [66.1,72.2] | 0.251 |
| Albumin |  | 40.6 [38.5,42.6] | 40.6 [38.3,42.6] | 40.7 [39.0,42.8] | 0.434 |
| Globulin |  | 27.8 [24.9,31.0] | 27.6 [24.6,31.0] | 28.1 [25.3,30.8] | 0.353 |
| Albumin/globulin ratio |  | 1.4 [1.3,1.6] | 1.4 [1.3,1.6] | 1.5 [1.3,1.6] | 0.726 |
| Creatinine |  | 72.6 [60.3,88.6] | 72.0 [60.1,87.6] | 73.2 [61.0,90.8] | 0.374 |
| Urea |  | 5.6 [4.4,7.3] | 5.4 [4.3,7.4] | 5.8 [4.5,7.1] | 0.152 |
| Uric acid |  | 328.1 [266.5,398.5] | 322.0 [265.2,393.2] | 336.0 [267.0,400.6] | 0.405 |
| HbA1c |  | 5.9 [5.5,6.6] | 5.9 [5.5,6.5] | 6.0 [5.6,6.8] | 0.069 |
| TC |  | 4.3 [3.7,4.9] | 4.2 [3.6,4.9] | 4.4 [3.8,5.1] | 0.228 |
| TG |  | 1.1 [0.8,1.6] | 1.1 [0.8,1.5] | 1.2 [0.8,1.6] | 0.455 |
| HDL-C |  | 1.1 [0.9,1.4] | 1.1 [0.9,1.4] | 1.1 [0.9,1.4] | 0.87 |
| LDL-C |  | 2.4 [1.9,3.0] | 2.3 [1.8,2.9] | 2.5 [2.0,3.0] | 0.08 |
| Lp(a) |  | 152.3 [79.1,315.7] | 148.9 [74.9,315.3] | 163.6 [84.1,315.9] | 0.453 |
| ApoA1 |  | 1.3 [1.1,1.4] | 1.3 [1.1,1.4] | 1.3 [1.1,1.4] | 0.717 |
| ApoB |  | 0.8 [0.7,1.0] | 0.8 [0.7,1.0] | 0.8 [0.7,1.0] | 0.207 |
| HCY |  | 15.8 [12.1,22.0] | 15.9 [12.2,22.0] | 15.7 [12.0,22.3] | 0.946 |
| CRP |  | 2.3 [1.0,6.6] | 2.0 [0.9,6.5] | 2.6 [1.1,6.5] | 0.202 |
| TBA |  | 3.9 [2.2,7.0] | 4.2 [2.2,7.2] | 3.7 [2.3,6.2] | 0.338 |
| Sodium |  | 139.7 [138.0,140.5] | 139.7 [138.0,140.6] | 139.6 [138.0,140.4] | 0.507 |
| Potassium |  | 3.7 [3.5,4.0] | 3.7 [3.5,4.0] | 3.7 [3.5,4.0] | 0.73 |
| Chloride |  | 104.7 [102.8,106.9] | 104.7 [103.0,106.9] | 104.4 [102.8,106.9] | 0.548 |
| Total calcium |  | 2.3 [2.2,2.3] | 2.3 [2.2,2.3] | 2.3 [2.2,2.3] | 0.52 |
| Magnesium |  | 0.8 [0.8,0.9] | 0.8 [0.8,0.9] | 0.8 [0.8,0.9] | 0.647 |
| Phosphate |  | 1.0 [0.8,1.1] | 1.0 [0.8,1.1] | 1.0 [0.8,1.1] | 0.745 |
| CK |  | 90.0 [60.0,138.0] | 91.0 [60.0,150.2] | 88.0 [60.0,129.7] | 0.586 |
| CK-MB |  | 21.1 [16.3,26.7] | 21.0 [16.2,26.3] | 21.2 [16.5,27.5] | 0.504 |

**Abbreviations and nomenclatures**

END, early neurological deterioration; ECG, electrocardiogram; PR, P-R interval; QRS, QRS complex duration; QT, QT interval; QTc, corrected QT interval; WBC, white blood cell count; RBC, red blood cell count; Hb, hemoglobin; HCT, hematocrit; MCV, mean corpuscular volume; MCH, mean corpuscular hemoglobin; MCHC, mean corpuscular hemoglobin concentration; RDW-CV, red cell distribution width-coefficient of variation; RDW-SD, red cell distribution width-standard deviation; PLT, platelet count; PCT, plateletcrit; MPV, mean platelet volume; PDW, platelet distribution width; P-LCR, platelet large cell ratio; PT, prothrombin time; PTR, prothrombin activity; INR, international normalized ratio; APTT, activated partial thromboplastin time; TT, thrombin time; Fbg, fibrinogen; D-dimer, D-dimer; ALT, alanine aminotransferase; AST, aspartate aminotransferase; GGT, gamma-glutamyl transferase; ALP, alkaline phosphatase; TC, total cholesterol; TG, triglycerides; HDL-C, high-density lipoprotein cholesterol; LDL-C, low-density lipoprotein cholesterol; Lp(a), lipoprotein(a); ApoA1, apolipoprotein A1; ApoB, apolipoprotein B; HCY, homocysteine; CRP, C-reactive protein; TBA, total bile acids; CK, creatine kinase; CK-MB, creatine kinase-MB isoenzyme; LDH, lactate dehydrogenase; HbA1c, hemoglobin A1c. Data are presented as mean (SD) or median [Q1, Q3].
